# Feasibility of deploying community health workers to assist with health-related social needs and hypertension in community care clinics

**DOI:** 10.1017/cts.2025.53

**Published:** 2025-03-31

**Authors:** Brian Robusto, Iris Cheng, Rohan Mahabaleshwarkar, Jessica McCutcheon, Nancy Denizard-Thompson, Sara R. Kinny, Selina Quinones, Henry Bundy, Yhenneko J. Taylor, Deepak Palakshappa

**Affiliations:** 1 Wake Forest University School of Medicine, Winston-Salem, NC, USA; 2 Department of Internal Medicine, Wake Forest University School of Medicine/Atrium Health, Charlotte, NC, USA; 3 Center for Health System Sciences, Atrium Health, Charlotte, NC, USA; 4 Department of Internal Medicine, Wake Forest University School of Medicine, Winston-Salem, NC, USA; 5 Department of Pediatrics, Wake Forest University School of Medicine, Winston-Salem, NC, USA; 6 Department of Epidemiology and Prevention, Division of Public Health Sciences, Wake Forest University School of Medicine, Winston-Salem, NC, USA

**Keywords:** Social determinants of health, health-related social needs, community health workers, hypertension, mixed methods, community primary care, health services

## Abstract

We conducted a pilot study of implementing community health workers (CHWs) to assist patients with hypertension and social needs. As part of clinical care, patients identified as having an unmet need were referred to a CHW. We evaluated changes in blood pressure and needs among 35 patients and conducted interviews to understand participants’ experiences. Participants had a mean age of 54.1 years and 29 were Black. Twenty-six completed follow-up. Blood pressure and social needs improved from baseline to 6 months. Participants reported being accepting of CHWs, but also challenges with establishing a relationship with a CHW and being unclear about their role.

## Background

Health-related social needs, such as food insecurity and housing instability, are strongly associated with worse health and impaired chronic disease management [1]. For example, people living with hypertension are more likely to have unmet health-related social needs, and unmet health-related social needs are associated with poor adherence to hypertension treatments (e.g., medications and a healthy diet) and worse blood pressure control [2–4]. National healthcare organizations, including the Centers for Medicare and Medicaid (CMS), have recommended that healthcare providers assess for and assist patients with health-related social needs [5,6]. One approach health systems are using to assist patients with social needs is to deploy community health workers (CHWs) [7]. CHWs are trusted members of the community, share a common background with patients, and assist individuals with accessing community services and provide social support [8]. CHWs have been employed in healthcare and public health settings to provide numerous types of services, including patient outreach, health education, and team-based care [7]. Several clinical trials have shown that CHWs are effective in addressing health-related social needs and improving health outcomes, but the impact and potential challenges of implementing CHWs in real-world settings are still unclear [7,9–12].

We conducted a mixed methods study at 4 community primary care clinics that had integrated teams of CHWs to assist patients with health-related social needs. Our objective was to determine the feasibility of patients being connected to a CHW in the participating clinics, patients’ acceptability of working with a CHW, and the potential effectiveness of deploying CHWs within the clinics to assist patients with unmet social needs and blood pressure management.

## Materials and methods

### Study design and population

Prior to the start of this study, each of the 4 clinics had integrated teams of CHWs to assist patients who were identified as having a health-related social need. All four clinics were affiliated with Atrium Health Greater Charlotte Region and primarily served populations with lower socioeconomic status and from historically marginalized racial and ethnic groups with high rates of chronic medical conditions, such as hypertension. The clinics are located throughout Greater Charlotte in diverse neighborhoods (Supplemental Fig. 1) and served diverse populations (Supplemental Table 1). We included these clinics because they were the first four clinics at Atrium Health that had integrated CHWs and the high number of patients with unmet health-related social needs.

**Table 1.** Characteristics of the program evaluation participants

	Baseline (N = 35)	3-month follow-up (N = 26)	6-month follow-up (N = 26)	p-value
Age, mean (SD)	54.1 (14.3)	–	–	–
Sex, N (%)				
Male	18 (51.4)	–	–	–
Female	17 (48.6)	–	–	–
Race, N (%)				
Black/African American	29 (82.9)	–	–	–
White	3 (8.6)	–	–	–
American Indian/Alaskan native	1 (2.9)	–	–	–
Other^a^	2 (5.7)	–	–	–
Ethnicity, N (%)				
Hispanic, Latino, or Spanish	3 (8.6)	–	–	–
Not Hispanic, Latino, or Spanish	32 (91.4)	–	–	–
Insurance, N (%)				
Private	6 (17.1)	–	–	–
Medicaid	4 (11.4)	–	–	–
Medicare	5 (14.3)	–	–	–
Uninsured	12 (34.3)	–	–	–
Other^b^	8 (22.9)	–	–	–
Charlson comorbidity index, mean (SD)	2.9 (2.8)	–	–	–
Number of blood pressure medications, mean (SD)	1.3 (1.2)	–	–	–
Systolic blood pressure, mean (SD)	161.2 (17.8)	145.1 (17.3)	147.4 (17.8)	<0.001
Diastolic blood pressure, mean (SD)	98.4 (17.0)	87.1 (15.2)	87.1 (15.4)	0.037
Hypertension self-efficacy, mean (SD)	58.7 (11.9)	58.7 (11.3)	58.9 (11.4)	0.35
Medication nonadherence, N (%)	14 (40)	12 (46.2)	11 (42.3)	0.24
Health-related social needs, N (%)				
Food insecurity	27 (77.1)	21 (80.8)	19 (73.1)	<0.001
Transportation	22 (62.9)	16 (61.5)	12 (46.2)	0.48
Housing	27 (77.1)	21 (80.8)	20 (76.9)	<0.001
Lack of companionship	24 (68.6)	15 (57.7)	18 (69.2)	0.05
Being left out	23 (65.7)	20 (76.9)	18 (69.2)	0.03
Feeling of isolation	23 (65.7)	17 (65.4)	20 (76.9)	0.03

^a^Includes Middle Eastern/North African, multiracial, and unknown; ^b^includes liability, sponsored agencies, Tricare, Worker’’s Compensation, and other governmental programs.

The CHWs were employed within the clinic and only worked with individuals who were patients at the clinic. The CHWs all received formal training through the University of Pennsylvania’s IMPaCT program and were certified through the North Carolina CHW training, which include training in working with medical providers, motivational interviewing, and de-escalating conflict [11,13]. As part of routine clinical care, all patients who presented for a visit were assessed for health-related social needs using the CMS Accountable Health Communities Health-Related Social Needs Questionnaire [6]. Patients identified as having an unmet need based on the questionnaire were referred by the clinician or staff to one of the clinic’s CHWs. If available, the CHW would meet with the patient in-person at the time of the visit to discuss the patient’s needs and assist with accessing community resources. If the CHW was not available at the time of the visit, she would contact the patient by phone. The CHW worked with the patient for at least 4 weeks and as long as 6 months based on patient’s needs.

We conducted a prospective mixed methods cohort study between 12/2022-4/2024. All adults (≥18 years) who had been referred to a CHW and had uncontrolled hypertension (defined as blood pressure >140/90 at the time of the visit) were eligible. After a potentially eligible patient was identified by clinic staff or a provider, a study team member contacted the patient, either in person or by phone, to discuss the study purpose and procedures, review eligibility criteria, and obtain informed consent. We limited this study to patients with uncontrolled hypertension because populations that have been socially and economically disadvantaged are at higher risk of uncontrolled hypertension and to determine the potential impact of assisting patients with social needs on blood pressure management. We excluded participants if they were unable to speak English or Spanish or had a severe cognitive impairment that would limit their ability to provide informed consent, and we enrolled 35 participants in the study. The Wake Forest University School of Medicine Institutional Review Board approved this study.

### Quantitative data collection

We collected blood pressure and survey data on participants at baseline, 3 months, and 6 months. All participants were provided an ambulatory blood pressure cuff and education on how to check their blood pressure using the cuff at the time of enrollment. The surveys (see Supplemental Tables 1 and 2) included questions on participants social needs [6], medication adherence (based on questions from the National Health Interview Survey), and hypertension self-efficacy (from the Hypertension Self-care Profile) [14]. We also assessed acceptability of working with the CHW using the Acceptability of Intervention Measure (AIM) [15]. Data collection occurred either in-person or by phone. We also collected demographic data on all participants through data extraction from the electronic health record and at the time of baseline survey collection. We only collected demographics at baseline to reduce the participants’ burden in completing the follow-up surveys and because the participant demographic characteristics collected for this study were unlikely to change over the 6-month period. Participants received a $20 gift card for each data collection timepoint (up to $60 total).

**Table 2.** Participant’s perspectives on working with a community health worker

*1. Challenges with managing blood pressure*	
Representative quotes	“My rent went up too much, and I have a hard time getting food. The food they give you from the food bank, it’s just like canned goods and stuff. And I don’t get the right meats or the protein I need for my body.”
	“I could just say my diet “cause I eat a lot of fast foods. I eat a lot of burgers. I ate a lot of red meat. I don’t really eat vegetables and fruits. Vegetable to me was French fries.”
	“It’s okay until I get stressed out [laughs], and then it goes up when I’m stressed.”

*2. Assistance provided by the CHW*	
Representative quotes	“[The CHW] encourages me to keep goin’ and keeps telling me to add more fruit, more water, more exercise. They’re all health related, encouraging conversations.”
	“She calls and talks to me in finding out things, how I am, and how things are going, and had I been writing down my blood pressure and stuff. And I told her, “Yes, that I was doing fine..” She just calls and makes sure there isn’t anything else I need. She talks to me and I enjoy her talkin’ to me.”
	“Because she [PCP provider] is a busy lady, and they [CHWs] communicate with her. I can communicate with my doctor, but the way they do it is better.”
	“I haven’t had anybody keeping up to make sure, you know how you say “keep up”? Keep up and say, “How did this go and how was this working? Did you get this email I sent you? I mailed you some new recipes and some things that might help out and stuff like that”…yeah, it’s been a whole lot better.”
	“She’s a sweetheart. She’s very nice. She’s very mannered and everything. I answer the questions that she has to ask. She calls me. And I appreciate that because—you know, I have a daughter around her age, too, so— well, my daughter might be a little older than her, but you know what I’m tryin’a say. So— I appreciate a young girl reachin’ out to me. That comforts me to know that somebody’s thinkin’ about me and see how I’m doin.’”

*3. Barriers to developing a relationship with the CHW*	
Representative quotes	“Well, I haven’t heard from her if she’s called me. And usually a lot people, you know, try to call me during the day when I’m at work. I’m always in front of people because of the job that I work. I work for the DMV, so I’ve constantly got people in my face and so I can’t answer my phone all the time. And if there’s not a message or even a text message or even an email, letting me know that you’re trying to call me. You know, I haven’t gotten any of that.”
	“I mean—well, I just had my phone cut back on, so that was one of ’em.”
	“Other than the fact of, you know, if you’re going to get in contact with a patient and you can’t get them via phone, you got the portal, but she never used the portal to reach out to me, my email address, or even text me.”
	“Most times [the CHW] called me, I just happened to be at work, and I don’t have a long time to talk to her like that.”

### Qualitative data collection

All participants were also offered the opportunity to participate in a semi-structured interview. To facilitate the interview, we developed an interview guide through a detailed review of the literature, consultation with outside experts, and input from our community advisory committee and clinical operations steering committee (see Supplemental Table 3). The community advisory committee consisted of 6 members who were either patients within the health system or members from local community organizations that assisted with social needs. Our clinical operations steering committee consisted of 10 health system or clinic leaders who had been involved in the integration of the CHW teams. The interview guide focused on 3 domains: 1) the effects of social needs on blood pressure control, 2) perceptions of working with the CHW, and 3) how to most effectively integrate CHWs. The guides were pilot tested for face validity with patients who were not included in the study.[16] Interviews occurred approximately 4-6 weeks after participants were referred to the CHW and lasted approximately 30 minutes. Participants received an additional $25 gift card for participating in an interview.

### Quantitative data analysis

We summarized patient characteristics using means and standard deviations (for continuous measures) and frequencies and percentages (for categorical measures). We used repeated measures ANOVA to evaluate the change in continuous outcomes (e.g., blood pressure) over time, and we used generalized estimating equations to evaluate change in categorical outcomes (e.g., social needs) over time. We used a 2-sided hypothesis test and considered an α <0.05 significant. We conducted all analyses using SAS version 9.4.

### Qualitative data analysis

All interviews were audio recorded, de-identified, and transcribed. The transcripts were then entered into ATLAS.ti software for data management and coding. We conducted a thematic analysis utilizing deductive and inductive coding, creating a codebook with definitions after initial review of all of the interviews, and then iteratively refined definitions and added codes as themes emerged throughout the coding process.[16] Two members of the research team independently applied codes and then came together to evaluate and compare coding. If there was not agreement, they discussed their perspectives and revisited the codebook until consensus was reached. Text segments were then reviewed by code and summarized. Code summaries were synthesized into themes and organized using the principles of reflexive thematic analysis.[17] We used triangulation with our community advisory committee and clinical operations steering committees to evaluate and establish the validity of the results.[16,17]

## Results

Of the 44 patients approached, we enrolled 35 (79.5%) participants with uncontrolled hypertension who had been referred to a CHW at one of the 4 clinics. This included 23 participants from clinic 1, 6 from clinic 2, 4 from clinic 3, and 2 from clinic 4. The mean age of participants was 54.1 years (SD 14.3 years), and the majority were male (51.4%) and Black (82.9%) (Table 1). Twenty-six participants (74.3%) completed 3-month and 26 participants (74.3%) completed 6-month follow-up. The mean AIM score was 4.2 (SD: 0.6). Both mean systolic (161.2, 145.1, and 147.4; *p* < 0.001) and mean diastolic (98.4, 87.1, and 87.1; *p* = 0.04) blood pressures were lower over time. However, we did not find a significant difference in hypertension self-efficacy or medication adherence. We found a lower percentage of participants reported concerns about food insecurity and lack of transportation over time.

Of the 35 participants, 27 completed an interview. We offered all participants the opportunity to schedule in an interview at the time of enrollment. Two participants reported they were not interested, and 6 participants were unable to be reached by phone for their scheduled interview. From the interviews, we identified 3 major themes. We provide representative quotations for these themes below, with additional supporting quotations in Table 2.

### Challenges with managing blood pressure

In most of the interviews, participants discussed the financial challenges with trying to manage their blood pressure. Many participants reported healthy foods were prohibitively expensive. “*The cost of food is definitely higher,*” Participant O said, “*…especially if you’re trying to eat healthier foods, it can be very expensive*.” For some, the difficulty of adhering to a healthy diet was compounded by the fact that a lot of the food they received from food banks was preserved, canned, and high in sodium. Others had difficulty finding transportation to doctors’ appointments or could not afford their medications. Still others struggled to meet basic needs and had little time to consider their health. “*I do my blood pressure when I think about it*,” Participant A said, “*cause I’m going through so much right now*.”

### Assistance provided by the CHW

Nearly every participant said they would recommend working with a CHW, who they characterized as helpful, encouraging, and competent. Participants reported that the CHW they worked with not only helped them in managing their blood pressure and other chronic medical conditions, but served as a liaison to clinicians and provided social support. Participant W noted, “[*My CHW] checks on my blood pressure, how my sugar is doing and we go over things that might help*.” Interviewee L, also said they found encouragement and motivation from their CHW, “*it makes me put more of an effort to do the things I need to do to get myself better*.” As noted in several interviews, often participants really appreciated that the CHW just showed concern for them, a rare quality according to Participant J, *“…there’s not too many people that do care*.”

### Barriers to developing a relationship with CHWs

Although most participants described the CHW they worked with as helpful and competent, some interviewees identified barriers to establishing a relationship with a CHW, including having a working phone and inflexible work schedules. “*On the days that we [the interviewee and the CHW] were supposed to meet*,” Interview G explained, “*I probably had no other choice but to work ’cause, that’s the only work I could get*.” Some participants also felt there was a limit to what their CHW could do. This rare complaint came from interviewees that wished that their CHW could be a little more hands-on. Many participants were also unsure of how closely their CHW worked with their primary care provider, and several participants said they were unclear of how their CHW fit into the organizational structure of the clinic.

## Discussion

In this study that evaluated the integration of CHWs in 4 community primary care clinics, we found that it was feasible to implement CHW teams to assist patients with unmet social needs, patients found it acceptable, and implementing CHW teams may be potentially effective in assisting patients with social needs and blood pressure management. Despite the potential benefits, patients noted several challenges to fully integrating CHWs in primary care settings. Similar to prior studies[7,18], these included logistical challenges with participants being able to establish a relationship with the CHW due to competing demands (e.g., patients having to work) and participants being unclear about the role of the CHWs within the clinic. Many participants also hoped the CHWs could have been able to provide additional assistance with both social needs (e.g., housing) and chronic disease management.

There are several limitations to this study. First, we only included 35 participants from 4 clinics that were affiliated with one health system, so the results may not be generalizable to other healthcare settings. Larger studies in the future are needed to understand the effectiveness and implementation of CHW teams in healthcare settings. Second, we focused on patient’s perspectives, further research is needed to understand CHWs and healthcare providers’ perspectives. Third, this was an observational study, so causation cannot be determined. Despite these limitations, this study provides important information on the feasibility and acceptability of implementing a CHW program in healthcare settings to assist patients with hypertension with unmet social needs.

## Supporting information

Robusto et al. supplementary material Robusto et al. supplementary material

## Supplementary material

The supplementary material for this article can be found at https://doi.org/10.1017/cts.2025.53.
