# Supplementary material for: Feasibility of deploying community health workers to assist with health-related social needs and hypertension in community care clinics
Source: J Clin Transl Sci. 2025 Mar 31;9(1):e81. doi: 10.1017/cts.2025.53 (PMC12083196; doi:10.1017/cts.2025.53)
Supplement: Robusto et al. supplementary material [file S2059866125000536sup001.docx]

**Supplemental Table 1: Demographics of the clinics include**

|  | **Clinic 1** | **Clinic 2** | **Clinic 3** | **Clinic 4** |
| --- | --- | --- | --- | --- |
| Patient Count | 5,125 | 7,739 | 2,140 | 7,531 |
| %Female | 59.5% | 61.6% | 69.3% | 61.7% |
| %Black | 50.0% | 75.4% | 36.8% | 61.4% |
| %Hispanic | 25.9% | 11.7% | 44.2% | 13.6% |
| %SelfPay | 22.1% | 9.8% | 24.9% | 9.5% |
| % Medicaid | 26.0% | 40.6% | 17.9% | 36.9% |
| %Medicare | 22.6% | 18.0% | 7.6% | 16.1% |

**Supplemental Table 2**: Participant Baseline Questionnaire

1. What is your housing situation today?

[ ] I do not have housing (I am staying with others, in a hotel, in a shelter, living outside on the street, on a beach, in a car, abandoned building, bus or train station, or in a park)

[ ] I have housing today, but I am worried about losing housing in the future.

[ ] I have housing

1. Within in the past 30 days, you worried that your food would run out before you got money to buy more.

[ ] Often true

[ ] Sometime true

[ ] Never true

1. Within in the past 30 days, the food you bought just didn’t last and you didn’t have money to get more.

[ ] Often true

[ ] Sometime true

[ ] Never true

1. In the past 12 months, has lack of transportation kept you from medical appointments, meetings, work or from getting things needed for daily living? (check all that apply)

[ ] Yes, it has kept me from medical appointments

[ ] Yes, it has kept me from getting medications

[ ] Yes, it has kept me from non-medical meetings, appointments, work, or getting things that I need

[ ] No

1. How often do you feel that you lack companionship?

[ ] Hardly ever

[ ] Some of the time

[ ] Often

1. How often do you feel left out?

[ ] Hardly ever

[ ] Some of the time

[ ] Often

1. How often do you feel isolated from others?

[ ] Hardly ever

[ ] Some of the time

[ ] Often

1. During the past 12 months, were you prescribed medication by a doctor or other health professional?

[ ] Yes

[ ] No

[ ] Unsure

1. During the past 12 months, were any of the following true for you?

- You skipped medication doses to save money. (yes or no)
- You took less medication to save money. (yes or no)
- You delayed filling a prescription to save money. (yes or no)

1. How many times in the past year have you used prescription drugs for non-medical reasons?

[ ] Never

[ ] Once or twice

[ ] Monthly

[ ] Weekly

[ ] Daily or almost daily

1. How many times in the past year have you used illegal drugs?

[ ] Never

[ ] Once or twice

[ ] Monthly

[ ] Weekly

[ ] Daily or almost daily

Hypertension self-care profile

Listed below are common recommendations for persons with hypertension. **How confident are you that you could,**

| **Items** | **Very confident** | **Confident** | **Somewhat confident** | **Not confident** |
| --- | --- | --- | --- | --- |
| C1. Take part in regular physical activity (example: 30 minutes of walking 4 to 5 times a week)? | _4_ | _3_ | _2_ | _1_ |
| C2. Eat less processed foods such as (e.g. lunch meats, canned or frozen foods)? | _4_ | _3_ | _2_ | _1_ |
| C3. Read nutrition facts labels to check information on how much sodium is in food? | _4_ | _3_ | _2_ | _1_ |
| C4. Replace high-salt foods (example: canned soups, Oodles of Noodles) with low-salt foods (example: homemade soups, fresh vegetables)? | _4_ | _3_ | _2_ | _1_ |
| C5. Limit use of high-salt condiments (e.g.ketchup) | _4_ | _3_ | _2_ | _1_ |
| C6. Eat less than 1 teaspoon of table salt per day (6 grams)? | _4_ | _3_ | _2_ | _1_ |
| C7. Eat less foods that are high in saturated fat (example: red meat, butter) and trans-fat (example: shortening, lard)? | _4_ | _3_ | _2_ | _1_ |
| C8. Use broil, bake or steam instead of frying when cooking? | _4_ | _3_ | _2_ | _1_ |
| C9. Read nutrition facts label to check information on saturated fat (example: butter, red meat) and trans-fat (example: lard, shortening)? | _4_ | _3_ | _2_ | _1_ |
| C10. Replace traditional high-fat foods (e.g. deep fried chicken) with low-fat products (e.g. baked chicken)? | _4_ | _3_ | _2_ | _1_ |
| C11. Limit total calorie intake from fat (less than 65grams) daily? | _4_ | _3_ | _2_ | _1_ |
| C12. Eat 5 or more servings of fruits and vegetables daily? | _4_ | _3_ | _2_ | _1_ |
| C13. Practice moderation in drinking alcohol daily (2 glasses or less for men; 1 glass or less for women)? | _4_ | _3_ | _2_ | _1_ |
| C14. Practice non-smoking? | _4_ | _3_ | _2_ | _1_ |
| C15. Check your blood pressure at home? | _4_ | _3_ | _2_ | _1_ |
| C16. Take your blood pressure medicine? | _4_ | _3_ | _2_ | _1_ |
| C17. Get your prescriptions filled? | _4_ | _3_ | _2_ | _1_ |
| C18. Keep your weight down? | _4_ | _3_ | _2_ | _1_ |
| C19. Try to stay away from anything and anybody that causes any kind of stress? | _4_ | _3_ | _2_ | _1_ |
| C20. See a doctor regularly? | _4_ | _3_ | _2_ | _1_ |

**Demographics**

Best number to reach you at:________________

1. How many people live in your home? ____________________
2. How many people under the age of 18 years live in your home? _______
3. Are you now married, widowed, divorced, separated, never married or living with a partner?
   1. Married
   2. Widowed
   3. Divorced
   4. Separated
   5. Never married
   6. Living with a partner
   7. Declined
4. What is the highest grade or level of school you have completed or the highest degree you have received
   1. Less than 9^th^ grade ______
   2. 9^th^-11^th^ grade______
   3. Graduated High school/GED or equivalent _______
   4. Some college ______
   5. College graduate or above _________
5. What was your total household income before taxes during the past 12 months?
   1. <20,000
   2. 20,000-40,000
   3. >40,000-75,000
   4. >75,000
   5. I prefer not to provide income information.

**Supplemental Table 3:** Participant Follow Up Questionnaire

1. What is your housing situation today?

[ ] I do not have housing (I am staying with others, in a hotel, in a shelter, living outside on the street, on a beach, in a car, abandoned building, bus or train station, or in a park)

[ ] I have housing today, but I am worried about losing housing in the future.

[ ] I have housing

1. Within in the past 30 days, you worried that your food would run out before you got money to buy more.

[ ] Often true

[ ] Sometime true

[ ] Never true

1. Within in the past 30 days, the food you bought just didn’t last and you didn’t have money to get more.

[ ] Often true

[ ] Sometime true

[ ] Never true

1. In the past 12 months, has lack of transportation kept you from medical appointments, meetings, work or from getting things needed for daily living? (check all that apply)

[ ] Yes, it has kept me from medical appointments

[ ] Yes, it has kept me from getting medications

[ ] Yes, it has kept me from non-medical meetings, appointments, work, or getting things that I need

[ ] No

1. How often do you feel that you lack companionship?

[ ] Hardly ever

[ ] Some of the time

[ ] Often

1. How often do you feel left out?

[ ] Hardly ever

[ ] Some of the time

[ ] Often

1. How often do you feel isolated from others?

[ ] Hardly ever

[ ] Some of the time

[ ] Often

1. During the past 12 months, were you prescribed medication by a doctor or other health professional?

[ ] Yes

[ ] No

[ ] Unsure

1. During the past 12 months, were any of the following true for you?

- You skipped medication doses to save money. (yes or no)
- You took less medication to save money. (yes or no)
- You delayed filling a prescription to save money. (yes or no)

1. How many times in the past year have you used prescription drugs for non-medical reasons?

[ ] Never

[ ] Once or twice

[ ] Monthly

[ ] Weekly

[ ] Daily or almost daily

1. How many times in the past year have you used illegal drugs?

[ ] Never

[ ] Once or twice

[ ] Monthly

[ ] Weekly

[ ] Daily or almost daily

Hypertension self-care profile

Listed below are common recommendations for persons with hypertension. **How confident are you that you could,**

| **Items** | **Very confident** | **Confident** | **Somewhat confident** | **Not confident** |
| --- | --- | --- | --- | --- |
| C1. Take part in regular physical activity (example: 30 minutes of walking 4 to 5 times a week)? | _4_ | _3_ | _2_ | _1_ |
| C2. Eat less processed foods such as (e.g. lunch meats, canned or frozen foods)? | _4_ | _3_ | _2_ | _1_ |
| C3. Read nutrition facts labels to check information on how much sodium is in food? | _4_ | _3_ | _2_ | _1_ |
| C4. Replace high-salt foods (example: canned soups, Oodles of Noodles) with low-salt foods (example: homemade soups, fresh vegetables)? | _4_ | _3_ | _2_ | _1_ |
| C5. Limit use of high-salt condiments (e.g.ketchup) | _4_ | _3_ | _2_ | _1_ |
| C6. Eat less than 1 teaspoon of table salt per day (6 grams)? | _4_ | _3_ | _2_ | _1_ |
| C7. Eat less foods that are high in saturated fat (example: red meat, butter) and trans-fat (example: shortening, lard)? | _4_ | _3_ | _2_ | _1_ |
| C8. Use broil, bake or steam instead of frying when cooking? | _4_ | _3_ | _2_ | _1_ |
| C9. Read nutrition facts label to check information on saturated fat (example: butter, red meat) and trans-fat (example: lard, shortening)? | _4_ | _3_ | _2_ | _1_ |
| C10. Replace traditional high-fat foods (e.g. deep fried chicken) with low-fat products (e.g. baked chicken)? | _4_ | _3_ | _2_ | _1_ |
| C11. Limit total calorie intake from fat (less than 65grams) daily? | _4_ | _3_ | _2_ | _1_ |
| C12. Eat 5 or more servings of fruits and vegetables daily? | _4_ | _3_ | _2_ | _1_ |
| C13. Practice moderation in drinking alcohol daily (2 glasses or less for men; 1 glass or less for women)? | _4_ | _3_ | _2_ | _1_ |
| C14. Practice non-smoking? | _4_ | _3_ | _2_ | _1_ |
| C15. Check your blood pressure at home? | _4_ | _3_ | _2_ | _1_ |
| C16. Take your blood pressure medicine? | _4_ | _3_ | _2_ | _1_ |
| C17. Get your prescriptions filled? | _4_ | _3_ | _2_ | _1_ |
| C18. Keep your weight down? | _4_ | _3_ | _2_ | _1_ |
| C19. Try to stay away from anything and anybody that causes any kind of stress? | _4_ | _3_ | _2_ | _1_ |
| C20. See a doctor regularly? | _4_ | _3_ | _2_ | _1_ |

**Acceptability of an Intervention Measure (AIM)**

|  | **Completely**  **disagree** | **Disagree** | **Neither agree nor disagree** | **Agree** | **Completely**  **agree** |
| --- | --- | --- | --- | --- | --- |
| 1. Working with the community health worker meets my approval. | 1 | 2 | 3 | 4 | 5 |
| 1. Working with the community health worker meets is appealing to me. | 1 | 2 | 3 | 4 | 5 |
| 1. I like working with the community health worker meets. | 1 | 2 | 3 | 4 | 5 |
| 1. I welcome Working with the community health worker meets. | 1 | 2 | 3 | 4 | 5 |

**Supplemental Table 4:** Patient Interview Guide

**Introduction [***Not to be read verbatim***]:**

Hi, my name is ________, and I am a researcher at Atrium Health. Thank you so much for agreeing to talk to me. The purpose of this interview is to hear about your experiences working with [name of CHW]. We want to understand how community health workers/patient coordinators, like [name of CHW], can better help patients, particularly with helping patients manage their blood pressure. In this interview I’d like to ask you some questions about your blood pressure and your work with [name of CHW]. Hearing your unique perspective is very important. Do you have any questions for me?

Ok, now, because this is a research study I am going to go over some information about what being in the study entails.

**Informed Consent**

Reminder: Informed consent must be obtained **BEFORE** starting the interview.

**Effects of social determinants of health on blood pressure control**

1. Tell me a little bit about when you were first diagnosed with high blood pressure?
2. How has it been managing your blood pressure?
3. What are some things that makes it hard to manage your blood pressure?
   1. Probe: What you eat
   2. Probe: Medications
   3. Probe: Social needs (e.g. having enough food, transportation)

**Perception of working with community health workers**

Over the last few weeks, you may or may not have been working [name of CHW]. We would like to learn more about your experiences with working with [her/him].

Did you ever meet with [name of CHW]?

If no-> next question

If yes-> skip to question 2

1. What were the barriers to establishing a relationship with the CHW? (for those patients who never met with the CHW; skip to next section)

2. Tell me how you first came to work with [name of CHW]?

3. What services does the CHW provide that you wouldn’t have gotten anywhere else?

1. Doctor (doctors office)
2. Family Member

4. Tell us about a typical session with [name of CHW].

- 1. Probe: Where do the sessions usually take place? (e.g. phone, in-person)
  2. Probe: How long do the sessions last?
  3. What are some of the things you discussed with [name of CHW] about your health? Your blood pressure?

5. In what ways has working with [name of CHW] been helpful?

- 1. Probe: Managing your health
  2. Probe: Managing blood pressure (e.g. education, medications)
  3. Probe: Social needs (e.g. food, housing, transportation)

6. Would you recommend working with CHW to a friend?

- 1. Why?
  2. What did you like most?
  3. Is there anything you wish was different?

7. What were some of the challenges of working with (name of CHW)?

**Effectively integrate community health workers with clinical teams**

1. Do you have a regular doctor you see or place you go for your blood pressure or other healthcare?
   1. If yes,
      1. How closely does [name of CHW] work with your regular doctors?
         1. Probe: Do you consider [name of CHW] a member of your healthcare team?
      2. Do you think it would be helpful to you if [name of CHW] worked more closely with your doctor?
         1. If yes
            1. Why?
            2. How do you think they could do that?
         2. If no, why not?
   2. If no, have you talked to [name of CHW] about finding a regular doctor or clinic?
      1. Probe: Why or why not?
      2. Probe: Would you want to talk to [name of CHW] about finding a regular doctor? Why or why not
2. Other than (name of CHW) and your doctor, who else do you work with to help manage your blood pressure?

Is there anything we didn’t talk about that you think is important for us to know or you would like to talk?

Thank you so much for answering our questions.

**Supplemental Figure** **1**: Location of clinics participating in the study

| 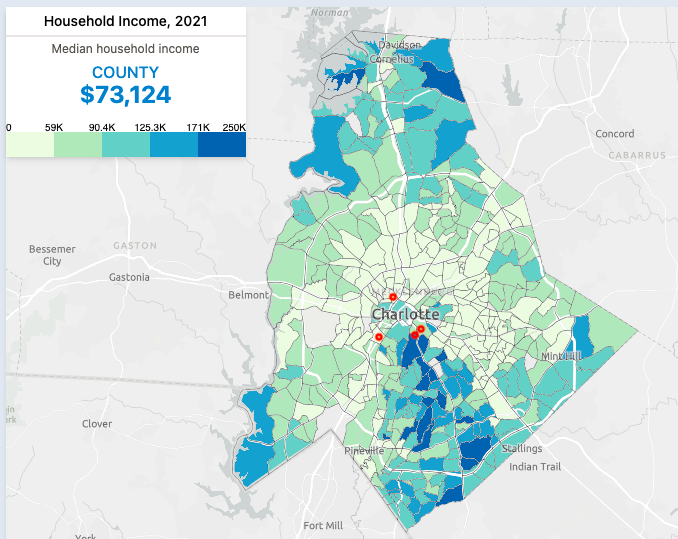 |
| --- |
